# Work Experience of Intensive Care Nurses during the Period of the Pandemic: A Qualitative Study

**DOI:** 10.4269/ajtmh.23-0136

**Published:** 2023-12-18

**Authors:** Gonca Karataş Baran, Sevcan Atasoy, Taşkın Tepe, Şenay Tanrıöver

**Affiliations:** ^1^ Intensive Care Clinic During Research, Ankara Etlik Zübeyde Hanım Women's Health Training and Research Hospital, University of Health Sciences, Ankara, Turkey;; ^2^ Training Nursing Department, Ankara Ataturk Sanatorium Training and Research Hospital, Ankara, Turkey;; ^3^ Intensive Care Clinic, Ankara Ataturk Sanatorium Training and Research Hospital, Ankara, Turkey;; ^4^ Health Care Services Department, Gulhane School of Nursing, University of Health Sciences, Ankara, Turkey

## Abstract

This study was conducted to describe the working experiences, feelings, and thoughts of nurses working in the intensive care unit (ICU) during the COVID-19 pandemic from their perspective. Empirical phenomenological approach, one of the qualitative study methods, was used to obtain detailed descriptions of the experiences of nurses working in the ICU during the COVID-19 outbreak in providing patient care. The research design is an in-depth interview technique. The Colaizzi method was used in the analysis of the interviews. The data obtained from the research were categorized in six main themes. These themes are as follows: backbone of the health system—nursing, professional achievements, difficulties encountered, support needs and expectations, changes in emotions, and private life. In the process of fighting the pandemic, ICU nurses undertook important responsibilities, quickly adapted to the crisis, and displayed a strong stance, thus carrying a huge burden within the healthcare system.

## INTRODUCTION

In the early stages of the pandemic in the world and in our country, many hospitals were converted into isolation hospitals. Healthcare providers began to serve patients with COVID-19 contacts during this period. In this process, while the countries of the world, including Turkey where this study was based, continued their fight against the pandemic, all health workers and nurses, who are at the forefront of care, took part in this difficult struggle without self-interest.^1^

Intensive care units (ICUs) have become the most important units in this infectious disease pandemic that has affected a large number of people.^2^ It has been shown that nurses experienced situations such as stress, anxiety, fear, uncertainty, and stigma during the pandemic period in many quantitative studies,^3^^–^^7^ but qualitative studies that examine the experiences of nurses in the event of a pandemic are limited worldwide and in Turkey.

In studies conducted during the COVID-19 pandemic, the psychological and emotional states in nurses,^8^^–^^10^ their professional experience,^8^^–^^10^ and their adaptation to the pandemic process^10^^,^^11^ were covered.

Phenomenological research focuses on experiences perceived or interpreted by participants. The Colaizzi method within the scope of phenomenological research focuses on deriving a collection of common attributes and themes from multiple responses rather than individual characteristics.^12^ In this study, we present the experiences, feelings, and thoughts of intensive care nurses working during the pandemic from their own perspectives. With this study, we reveal the unrecognized problems of intensive care nurses during the pandemic period. With this research, we enabled nurses to express themselves. In addition, this study will provide a guide in providing a better quality care environment with an in-depth understanding of the nursing experience as told by nurses during the pandemic process and the determination of the needs of nursing services.

## MATERIALS AND METHODS

This study was conducted to describe the working experiences, feelings, and thoughts of nurses working in the ICU during the COVID-19 pandemic from their own perspectives.

An empirical phenomenological (descriptive phenomenology) approach, one of the qualitative research methods, was used to obtain detailed descriptions of the experiences of nurses working/working in intensive care in providing patient care during the COVID-19 pandemic. The research design was an in-depth interview technique.

### General environment, patient population, and staffing levels.

The research was conducted between July 15, 2020 and March 15, 2021. The population of the study consisted of nurses who previously worked or were currently working in the COVID ICU at one hospital. A similar sampling method, the purposive sampling methods, was used to determine the sample size, and saturation was taken as the basis for the sample size. In the research, in-depth interviews were conducted with 50 nurses using semistructured questions. None of the participants refused to participate in the study.

### Data collection.

All interviews were audio-recorded with the permission of the participant. Demographic data were collected. Interviews were conducted by researcher nurses (one PhD intensive care nurse (female), one intensive care nurse (male), one executive nurse (female), and one training nurse (female). The interviews lasted an average of 20 minutes, and data were collected individually in the clinic. Audio-recordings of the interviews were made.

Semistructured research questions were as follows:

- “Can you tell us about your experience caring for COVID-19 patients?”
- “What are the differences between working during the pandemic and working before the pandemic?”
- “How did you feel while working in the first days of the pandemic? What changes did you have in your emotions as the process progressed?
- “What difficulties did you encounter while working during the pandemic period? What did you do to overcome these difficulties?
- “What kind of support did you need while working during the pandemic period?”
- “What effects did working during the pandemic have on your family/private life?”

To produce comprehensive data, the statements were explained and deepened with open-ended question types according to the flow of the interview.

### Data analysis.

**Figure 1.**
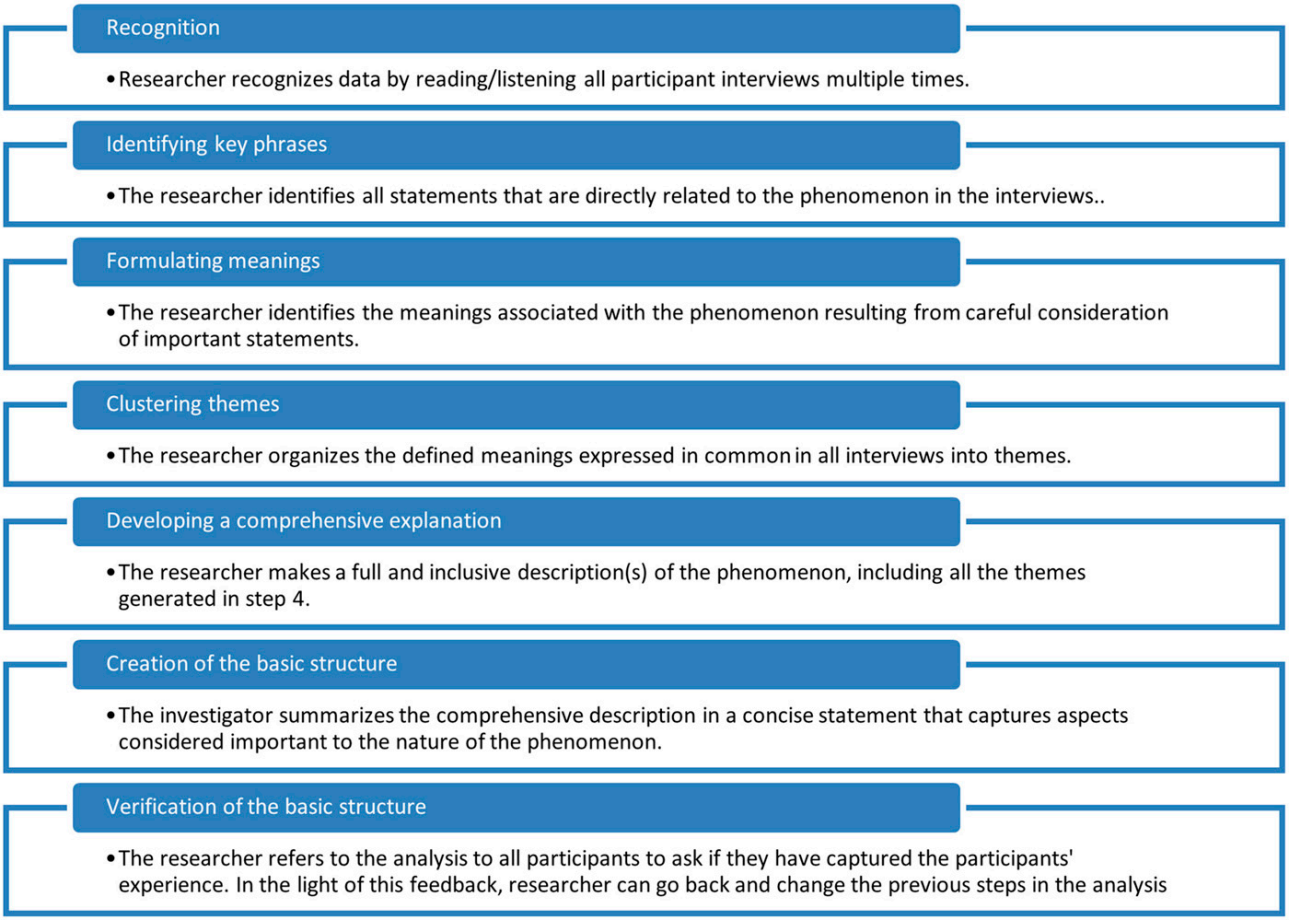
Colaizzi descriptive phenomenological method.

Analysis of descriptive (sociodemographic) data was done using SPSS 22 (IBM, Armonk, NY). The Colaizzi method was used in the analysis of the interviews. Colaizzi’s (1978) distinctive seven-step process provides a rigorous analysis that includes each step. The final result is a short and concise explanation of the text (Figure 1).^13^

Individual data analysis was carried out simultaneously with the collected data. The audio-recordings were analyzed by the researchers (at least two nurse researchers) within the first 24 hours after the interviews, and the results of the joint analysis were confirmed by the researchers in terms of accuracy by interviewing the participants. During the main data analysis, all authors selected the cited quotations that agreed with the results, and the overall data analysis was carried out in the light of the results that were agreed on by all the authors. The themes were generated from the data. Results were categorized according to qualitative research reporting criteria (COnsolidated criteria for REporting Qualitative research [COREQ]).

The reliability of the research was provided by credibility, transferability, dependability, and confirmability criteria. A well-known research analysis method was used for credibility: two of the researchers were people who had long-term contact with the participants and had observations in the work environment, based on volunteerism, reliability between coders. All researchers took part in the general analysis processes. For reliability, the audit trail and step-by-step repetition techniques were used. In accordance with the audit trail technique, the data were recorded and stored to ensure that all analysis steps could be traced back to the original interviews. In accordance with the step-by-step repetition technique, at least two researchers analyzed and compared the data. For confirmability, feedback and confirmation interviews were conducted with at least two researchers. For transferability, participant characteristics and the methods and techniques used were clearly described in the publication of the research. Adequate citations collected through in-depth interviews were identified and included in the publication.

## RESULTS

The mean age of the participants was 31.36 ± 6.76 years; the majority were university graduates and experienced nurses. Most of the nurses had ICU experience in the pre-pandemic period. They worked in the COVID ICU for an average of 8 months, and half of the participants had had COVID infection (Table 1).

**Table 1:** Sociodemographic characteristics of the participants

Sociodemographic characteristics	*n*	%	Mean ± SD
			Med (min–max)
Age	50		31.36 ± 6.76
			30 (22–44)
Educational status			
Associate	3	6.0	
University	42	84.0	
Master	5	10.0	
Years of professional work			
0–1	9	18.0	
> 1–3	5	10	
> 3–5	5	10	
> 5–10	7	14	
> 10–20	21	42	
> 20	3	6	
Marital status			
Married	29	58	
Single	18	36	
Reserved/other	3	6	
Has child(ren)			
Yes	27	54	
No	23	46	
Income status			
Income less than expenses	10	20	
Income equal to expenses	29	58	
Income more than expenses	23	46	
Family type			
Core	47	94	
Wide	3	6	
Pre-Pandemic working unit			
Adult ICU	37	74	
NICU	4	8	
Never worked before	7	14	
Other	2	4	
Working time in the ICU before the pandemic			
Adult ICU + NICU time worked in the past (months)	43		63.93 ± 58.43
			48 (1–216)
COVID ICU working time (months)			7.96 ± 3.45
			8.5 (1–13)
COVID disease status			
Had	27	54	
Had not had	23	46	

ICU = intensive care unit; NICU = neonatal intensive care unit.

The data obtained from the research were categorized into six main themes: backbone of the health system—nursing, professional achievements, difficulties encountered, support needs and expectations, changes in emotions and private life (Table 2).

**Table 2:** Theme categories

1. The backbone of the health system - Nursing	4. Support needs and expectations
1.A. To shoulder the burden of the pandemic process	4.A. Need to be understood, psychological support
1.B. COVID heroes	4.B. Personal hygiene facilities
1.C. Key role in multidisciplinary approach	4.C. Fair remuneration, reward
1.D. Conscientious obligation	4.D. Flexible working arrangement
1.E. Providing quality, effective care	4.E. Qualified health personnel
1.F. Professional satisfaction	4.F. Giving the necessary value to the profession
2. Professional achievements	4.G. Desire to see positive results of care
2.A. Experience	4.H. Clarified job description
2.B. Professional care	4.I. Branching
2.C. Crisis management	5. Change in emotions
2.D. “Illness” point of view instead of “disease”	5.A. Stress, worry, fear, doubt, uncertainty, tension
2.E. Increase in empathy ability	5.B. Feeling of failure, sadness, unhappiness, low mood
2.F. Recognition of professional value	5.C. Feeling of deprivation, loneliness
3. Difficulties encountered	5.D. Alienation from the profession, decrease in the desire to work
3.A. Working with protective equipment	5.E. Trauma
3.B. Close contact with patients	6. Changes in private life
3.C. Increase in physical complaints	6.A. Childcare needs
3.D. Adaptation issues	6.B. Decrease in social activity, monotonous-isolated life
3.E. The marginalization of healthcare workers	6.C. Fear of death
3.F. Uncertainty about the process	6.D. Using technology actively

The first theme in our study is the backbone of the health system—nursing. They stated that the participating nurses shoulder the burden of the pandemic process and that they play a key role in the multidisciplinary approach. The nurses stated that they felt a conscientious obligation during the pandemic process, and that they achieved professional satisfaction by providing quality and effective care.

*This was a war and the nurses also took part in the war. (P20, P39)*

*We worked for the national mission. (P14)*

*Just like in the war, we shielded our body against bullets and we continue to do so. (P6)*

*Healthcare workers shouldered the heavy burden in this process, especially intensive care workers bear the heavy burden of the pandemic. (P10)*

The second theme, determined according to the opinions of the participants, focused on the “professional achievements of nursing during the pandemic process.” In this process, nurses encountered special situations and treatment protocols for COVID-19, different from the patient profile they care for, experienced different care methods, and gained practicality in care practices.

*We were practicing the profession we knew, managing a process we did not know. (P1)*

*We witnessed a sudden change in prognosis in this disease. We had fears and shyness before, but then we got over it. We have confidence in ourselves. We have improved ourselves in coping with this disease. (P18)*

Nurses stated that they did not compromise on professional care in this process, despite the risk of contagion, that they adapted quickly to this process, improved their crisis management skills, and were more successful in planning and organizing their work. They stated that they gained the ability to cope with this process by providing special infection control to the pandemic process, specializing in the care and follow-up of critical patients.

*The pandemic was difficult, but it had its rewards. It’s been an important experience. We learned more professional intervention to the patient with respiratory distress. We witnessed the different formations of the process. (P27)*

Nurses stated that during this process, their empathy skills increased, their emotional support roles developed, they gained a patient perspective instead of illness, and their professional values were noticed.

*Our job is human life, whether it’s a pandemic or not, the most important thing is to do what is necessary with compassion, the best thing we can do is to be with the patient. (P5)*

*The bridge between us and the patient has been shortened during this pandemic process. I felt that I was approaching patients. I felt like a hand was extended to me, waiting to be held. (P25)*

In the third theme, “difficulties encountered,” during the pandemic process were defined. Nurses stated that they work in close contact with patients for a long time and therefore the risk of infection is high. They expressed the feeling of discomfort caused by working with protective equipment.

*We worked as if we were hugging the patient. (P36)*

*In terms of care, I can say the same for before and after the pandemic. But more frequent aspiration, more oral care, positioning, drug application etc. are done more frequently. Patients have severe respiratory distress. The difficulties of working with protective equipment were among the additional burdens. (P34)*

The lack of knowledge and experience regarding the COVID disease process, the need for trained intensive care nurses, and the fact that nurses with no experience have to work in the intensive care have emerged as difficulties that increase the workload both in patient care and in sharing information of nurses.

*It was difficult to work with a team that was just starting out, did not know intensive care, and did not know how to work in the pandemic. In addition to our heavy workload, we also took on the role of providing training to our nurse friends who do not have intensive care experience. (P28).*

Nurses working during the pandemic were physically uncomfortable from regularly undergoing polymerase chain reaction swabs, and being infected with COVID was a very challenging psychological situation, both for them and their families. It took time for them to return to their duties in a healthy way. The fact that they were socially excluded in the whole process caused them to feel excluded, and they were psychologically worn out.

*Healthcare workers were perceived as individuals spreading COVID-19. (P3)*

*I was very afraid of being infected and going through the process that patients went through. (P20)*

The fourth theme, in the category of “support needs and professional expectations,” we first wanted to understand the experiences of nurses during the pandemic process and the need to provide psychological support to them and their families.

*The care was always the same, but the stress of the pandemic tired us out. I guess that’s burnout. It’s also hard not to know how long it will take. I want to work in intensive care as before. Not being able to see the results of my efforts no longer gives me pleasure in my work, it breaks my motivation. (P44)*

*We need psychological support. (P3, P27, P40, P33)*

Participants expressed that they wanted to receive attention and motivation support from the hospital management they work for, as well as suitable resting environments to be created in the work environment.

*I would like to be completely cleaned out of the ICU environment, I would like shower facilities to be provided. Our rest area was in intensive care before. Resting in an infected environment was uncomfortable. Afterward, the rest area provided outside the environment made us very comfortable. (P31)*

The participants stated that the importance of the professional values of nurses should be understood, that they deserve more pay in the face of the responsibility they take, and that high motivation can be achieved by noticing their efforts. They stated that a flexible working arrangement is necessary to ensure that they stay away from the work environment sufficiently and as much as necessary during the pandemic process and that this can only be possible with the presence of qualified health workers in hospitals. The nurses also appreciated the various support opportunities provided (e.g., the possibility of staying in guesthouses, free public transport).

*We want our ministry to give full support to health workers. If not now, when will the value of health workers be understood? (P39)*

*The work we do is too valuable to be measured in material terms (P3)*

They wanted the job descriptions of the nursing profession to be clear in every working environment, and they believed that the limits of their work would bring professionalism to their profession. They emphasized that as the need for intensive care nurses was seen to be high in the pandemic, and thus more nurses who can work in special units should be trained. In this regard, they said that applied nursing education should be given importance, and the training of medical devices used in the working areas should be given regularly.

*I wish our job descriptions were clear, in the current situation it is very open-ended. I do things that others don’t want to do. The workload is always on us nurses. (P34)*

*Someone put us in intensive care, we just work, no one sees or hears us. We will always try. Everything is expected from nurses. It is not clear what to do, you do things that are not your duty, you make up for the deficiencies. (P49)*

*We need colleagues who bring life to life. It is necessary to train qualified nurses with emphasis on applied education. (P7)*

As a result of the care they gave to the patients, the nurses wanted to see the patients who were taken to the service from the ICU and were discharged after recovery. When they saw these patients, they were happy, their motivation increased, and they felt a great relief.

*The loss of young patients has worn us out. We were happy because of the discharged patient. (P15)*

Team communication and support among their colleagues made them not feel alone, and they also met their support needs by overcoming difficulties with the sharing of knowledge and experience among them.

*We have always supported each other in this process. (P17)*

*We have team spirit. (P3)*

In the fifth theme of our study, in the category of “change in emotions”, nurses stated that they had experienced various mood changes (stress, fear, anxiety, uneasiness, doubt, sense of failure, sadness, grief, restlessness, depression, unhappiness, demoralization) since the beginning of the pandemic.

*At the beginning of the pandemic process, there was anxiety, uneasiness, and fear. (P44)*

*My initial anxiety and stress are no longer there. But the unknown still continues. (P30)*

*I’m worried. Because there is direct contact. I work with close contact for approximately 1.5 hours in every care of me. (P40)*

The fact that the uncertainty about the pandemic did not disappear, the state of constant protection caused agitation and nervousness from time to time, and this situation negatively affected their workplace and family life. Being apart from family members was the most important reason for the feeling of loneliness along with the feeling of longing.

*The workplace became our social environment. (P9)*

*We are very upset that healthcare workers are treated like they have the plague. (P3)*

*We are isolated from society. Even greetings were not given. (P27)*

With the ongoing pandemic process, nurses accepted the situation over time and adapted to the process and what it brings. They found meaning from their experiences, provided knowledge transfer, and are proud of themselves for their courage and potential to overcome difficulties.

*I always think, how can I be better protected, how can I protect myself? (P3)*

*We touched everything about life with the experience we gained. (P9)*

*Right now we see COVID as a normal disease. There is no difference except for personal protective equipment. (P19)*

The sixth theme was in the category of changes in private life, and the changes experienced by nurses in their private lives during the pandemic were discussed. They experienced restrictions in their social activities due to the fear of infection, led a monotonous and isolated life, and had to restrict their friends and family relationships. During the pandemic process, the caregivers of the children of the nurses could not continue to work, so they needed a caregiver and a nursery. They have experienced COVID losses in their first-degree relatives, and they felt the fear of death more intensely.

*Being separated from my child was traumatizing for me. Our post-shift cleaning process was also taking a long time. I even thought of burning the jerseys so as not to dirty the washing machine. (P30)*

*I realized the value of time spent with my loved ones. I realized that life is actually short and how important it is to live and live in the moment. (P50)*

## DISCUSSION

The importance of the concept of “care,” which is the main purpose of nursing, has emerged due to the COVID-19 pandemic and the fact that many patients became infected in a short time and needed intensive care. Nurses have an important place in the prevention, management, and reduction of infectious diseases such as COVID-19, which ranks third among the causes of death (according to the CDC death report 2021)and in the education of the society.^14^ At the end of our study, it was seen that the nurses working in the ICU during the pandemic period, were aware of their professional responsibilities in this struggle and worked hard to protect the whole country from the pandemic with the awareness of “a patriotic duty” without any self-interest. Considering the acute emergence of the COVID-19 pandemic and the insufficient number of intensive care nurses, it has been observed that intensive care nurses successfully managed the process with self-sacrifice. The WHO director-general emphasized the importance of nursing with the statements “Nursing in a country should be seen as a health investment, not a cost” and “Nurses are the backbone of the health system and are at the forefront of the fight” against COVID-19.”^1^

In the report created by the International Council of Nurses with the data collected from the member countries, it was emphasized that 20% of nurses were infected, and the extent of the risk and danger that nurses took during the pandemic process was strikingly clear.^15^ Bassett and Stanley stated in their study that nurses, together with other caregivers, worked like folk heroes and saviors during the COVID-19 pandemic process, and some nurses unfortunately lost their lives as a result of providing care.^16^ In our study, it was determined that half of the participants were infected with COVID. For these reasons, professional risks await nurses to provide care during the COVID pandemic in clinics or in all public units where people are present.

Nurses who provide professional care have managed to adapt to the pandemic process in a short time and continue their services safely and in accordance with individual needs.^17^ The nurses interviewed in our study have encountered specific cases and treatment protocols for COVID-19, different from the patient profile they have given care to date.

Nursing care provides not only physical but also psychological care and support in the fight against COVID, as in past infectious diseases. During the pandemic, nurses were often with them in the last moments of their lives. Even in the post-mortem period, nursing care continues by making preparations for the transportation of the patient, ensuring proper transportation, and supporting the mourning process of his relatives.^18^

The COVID-19 pandemic has significantly affected nursing services management and patient care processes, and as a result of the increase in the number of critical patients and the rapid increase in the needs to be met, the qualified human resources crisis has increased the workload of all health professionals, especially nurses.^19^ Intensive care units is among the units with high viral load due to aerosol-generating interventions, such as intubation, bronchoscopy, and aspiration.^20^ Lucchini et al. stated that the increase in the number of intensive care patients with the pandemic caused a 33% increase in the nursing workload.^17^ As stated by the participants in our study, the lack of knowledge and experience regarding the COVID disease process, the need for trained intensive care nurses, and the fact that nurses with no experience had to work in the ICU are the difficulties that increase the workload of both patient care and nurses in sharing information. Nursing workforce and support systems need to be well planned for quality and safe care. The participants in our study and Bambi et al. statedthat the most important lack of resources in the COVID-19 pandemic is “competent people” and that manpower is important in the fight against the pandemic.^19^

In this process, nurses who are closely involved in the care of the patient encounter physical and psychosocial problems due to reasons such as the risk of exposure to the virus, as well as the working environment, long and fast-paced working hours, and experience great difficulties with the fear of transmitting the virus to someone else.^21^ It was reported that health workers were adversely affected by this process during pandemic periods, and in case the need for health workers increased, there was an increase in the level of reluctance and anxiety in individuals.^22^ In a study conducted in China, it was shown that 8% of healthcare professionals considered quitting their job during the COVID-19 pandemic.^23^

Working actively in pandemics is a challenge in itself. In many studies, it is stated that being a healthcare worker in an pandemic creates a high level of biopsychosocial stress, even if it is not traumatic.^4^^–^^6^ Serrano-Ripoll et al., who investigated the effects of pandemics on the mental health of healthcare workers, found that health professionals both during and after pandemics reported high levels of acute stress disorder, anxiety, burnout, depression, and posttraumatic stress disorder.^24^ In our study, nurses stated that they experienced negative emotions. It has been reported that this situation arises from facing the death of the patients they care for, increased workload, threat of infection, and concern for their families.^25^ Liang et al., in contrast, reported that high levels of depressive symptoms were observed especially in nurses and physicians working in ICUs.^26^

Given the uncertainty about the pandemic process, the work and family lives of nurses have been adversely affected. This situation caused alienation from the profession and a decrease in their desire to work, and they were psychologically shaken by the effect of the trauma they experienced. In the 2020 study of Kıraner et al., it was observed that intensive care nurses experienced serious stress and anxiety for themselves, their families, and their immediate surroundings.^27^ Intensive care services are a stressful and isolated environment, and the pressure of care and work also affects the job satisfaction of the nurses working in these units.^28^ Intense working conditions and excessive workload are associated with patient safety and poor quality of life in nurses. This may cause performance problems in nurses.^13^

Healthcare workers are worried about the possibility of infecting their relatives.^2^ For these reasons, healthcare professionals prefer to stay away from their family members for long periods of time. These processes undoubtedly lead to a significant decrease in the emotional and social support provided by the family. This situation has been experienced frequently during the fight against COVID-19.^3^

The quality of life of healthcare professionals significantly affects the quality of care they provide.^29^ Mohindra et al. emphasized that the motivation levels of health professionals, including nurses, should be strengthened, that health workers should be focused on proper nutrition and rest.^30^ The tools that health professionals say are useful in the fight against the stressors accompanying the pandemic process include active coping (directly targeting the problem), positive reframing, social support from colleagues and family, and positive responses and support from hospital management.^31^^,^^32^

We note that a limitation of this study is that results are limited to the opinions of the participants.

## CONCLUSION

Our study shows that nurses play a vital role in the provision of health services. In the process of fighting the epidemic, intensive care nurses assumed vital responsibilities; adapted quickly to the crisis by using their critical thinking, problem-solving, creativity, and decision-making skills; and displayed a strong stance, thus carrying a huge burden within the healthcare system. As we are struggled and continue to struggle with COVID-19, nurses who do their profession with great devotion and self-sacrifice, as always, earn the respect of the whole world. It is vital that health systems are aware of the central role of nurses and invest in sustaining the success achieved by their dedication and effort during the pandemic, improve nurses’ personnel rights, and clarify action plans to acknowledge their contributions and duties.
